# The impact of agglomeration economies on hospital input prices

**DOI:** 10.1186/s13561-015-0075-1

**Published:** 2015-12-08

**Authors:** Andrew I. Friedson, Jing Li

**Affiliations:** 1 Department of Economics, University of Colorado Denver, 1380 Lawrence Street, Suite 460F, Denver, USA; 2 School of Economics, Singapore Management University, 90 Stamford Road, Singapore, 178903 Singapore

**Keywords:** Agglomeration, Health care, Input sharing, R00, I11, L23

## Abstract

This paper examines the extent to which agglomeration of the hospital service industry enhances the productivity of producing health care. Specifically, we use a large set of private insurance claims from the FAIR Health database to show that an increasing spatial concentration of hospital services results in a decreased cost of obtaining intermediate medical services. We explicitly test whether the reduced cost at concentrated locations arises from the ability to share intermediate service providers. The identification relies on state variation in medical lab technician licensure requirements, which influence the cost of intermediate services only through the cost of running a lab. Our findings suggest that agglomeration of the hospital service industry attracts specialized medical labs, which in turn help to reduce the cost of producing laboratory tests.

**Electronic supplementary material:**

The online version of this article (doi:10.1186/s13561-015-0075-1) contains supplementary material, which is available to authorized users.

## Background

Health care resources are highly concentrated in the United States. As documented by the Dartmouth Atlas of Health Care, a large fraction of hospital beds are located in a small number of areas, including California, the Chicago area, the Northeast coastal area, and Florida.^1^ The tremendous concentration of health care capacity in a small number of locations has received much attention in recent years, especially with respect to whether health outcomes have been sufficiently improved in areas concentrated with a large amount of medical resources.^2^ In contrast, less attention has been paid to the potential cost savings that are likely generated by agglomeration and related spillover effects arising from spatial concentration of medical resources. This paper explores the extent to which local agglomeration of medical services fosters spillover effects that enhance the efficiency with which medical services are provided and which further leads to a reduced cost of producing health care. Understanding the impact of hospital concentrations on cost savings is valuable for both U.S. health policy and hospital administration going forward as U.S. hospitals face increasing pressure to operate more efficiently.

It has long been recognized in the urban economics literature that productivity is often enhanced when firms operate in concentrated locations.^3^ Firms which locate near each other benefit from external economies of scale also known as agglomeration economies.^4^ These benefits arise from various channels. For instance, firm clusters facilitate the spread of knowledge due to increased opportunities for learning from nearby workers and firms (i.e. knowledge spillovers); clusters of firms also gain the advantage of drawing upon skilled pools of nearby labor (i.e. labor market pooling) as well as the ability to share valuable intermediate input providers (i.e., input sharing).

There is a large empirical literature on the nature and magnitude of external economies of scale, especially on the subject of how they arise through the channels of knowledge spillovers and labor market pooling.^5^ For the health care industry in particular, Bates and Santerre [1] showed that a 10 % increase in the number of hospitals per capita is associated with a 4.6 percent increase in hospital productivity. These benefits to hospitals from spatial concentration have been shown empirically to come from labor market pooling, which provides better job matches, and from knowledge spillovers, which increase the efficiency of care [2].

There is a comparatively smaller empirical literature studying agglomeration benefits through the sharing of intermediate input providers, which this study will contribute to. Holmes [3] demonstrated that industries with a greater degree of localization also tend to have a greater degree of vertical disintegration. This result was further reinforced for the health care sector by Li [4], who showed that hospitals in agglomerated areas are more likely to outsource intermediate services such as laboratory tests. Both studies documented the linkage between agglomeration economies and outsourcing behavior. One key result still missing from this literature is an estimate of the magnitude of cost savings or productivity gains that hospitals (or firms in general) reap from sharing intermediate input providers in concentrated locations.

This study extends the literature by examining how agglomeration of the hospital service industry enhances the productivity of producing health care through the channel of input sharing. Specifically, we examine the magnitude to which increasing spatial concentration of hospital services results in a decreased cost of obtaining intermediate medical services, in this case laboratory tests.^6^ We explicitly test whether the reduced cost of lab tests at concentrated locations arises from the ability to share intermediate service providers by drawing on state variation in medical lab technician licensure requirements that exogenously impact the cost of running a lab. Because state licensure requirements likely affect the cost of intermediate medical services only through the cost of running a lab, we are able to use this variation in licensure requirements as an instrument to identify whether agglomeration of the hospital service industry attracts specialized medical labs, which in turn help to reduce the cost of producing laboratory tests.

Our findings contribute to both the health economics literature and the urban economics/agglomeration literature. They contribute to the health economics literature by emphasizing a relatively new perspective in health economics – external economies of scale, and specifically input sharing – for improving the productivity of health care services. This is especially important for the hospital industry as controlling costs has become a critical issue not just for individual hospitals, but for the society in general. Our findings also contribute to the agglomeration literature by providing evidence that intermediate input sharing is a significant contributor to agglomeration benefits. This is the first estimation of the exact magnitude of cost savings associated with input sharing, which has to this point been a missing piece of the literature.

To achieve the aforementioned goals, we first present a model of hospital outsourcing behavior for intermediate medical services. The model has two main predictions that we later empirically test. First, when the concentration of hospital services increases, the size of the intermediate input industry (i.e., independent medical labs) should also increase. Second, when the concentration of hospitals increases, the price of intermediate services should then decrease.^7^ Because both the price of the intermediate service and the size of the intermediate industry are simultaneously determined and as higher prices may reversely lead to more employment in local medical labs, we need an instrument to identify what portion of the price decrease is due to the increase in the size of the input industry. The model suggests that an ideal instrument is the cost of entry for intermediate input providers, in the sense that it affects the price of intermediate medical services only through its deterrence of the entry of independent medical labs. In practice, it is extremely difficult to find a measure of entry costs for independent labs that is not also a measure of costs for in-house labs. As an alternative instrument, we use variation in state licensure requirements for medical laboratory technicians. State licensure requirements make hiring technicians more expensive, and only impact the price of intermediate services through the cost of running a lab. In this sense, differences in licensing requirements provides an exogenous shock, which could not be the result of reverse causality, to the cost of providing intermediate medical services.

We empirically test the model using data compiled from several data sources. Data on the price of laboratory tests come from the restricted FAIR Health Medical/Surgical database. The FAIR Health data are merged with hospital concentration information from the Centers for Medicaid and Medicare Services Provider of Services files, and with population counts and demographics information from the U.S. Census and the Area Resource File. The final data source is the Dun and Bradstreet database from which we acquire information on independent laboratory employment. Empirical findings show that the lab technician licensing requirements reduce the size of medical labs, which further lead to an increasing in the cost of performing various tests. The evidence is consistent with our model predictions.

The rest of the paper proceeds as follows. Section 'Model' discusses the model that generates two hypotheses to be tested in the empirical section. Section 'Data' describes the data and state specific lab technician licensing laws. Section 'Methods' discusses the identification strategy and empirical specifications. Results are presented in Section 'Results'. Section 'Discussion' concludes.

## Model

Agglomeration of the hospital service industry reduces the price of intermediate medical services through two channels. The first is that concentration of the medical industry promotes the entry of intermediate medical service suppliers into the local area and the increased number of intermediate suppliers then results in lower prices for intermediate goods due to increased competition. This is pecuniary agglomeration economies as in Brueckner [5] and it does not involve the increasing returns to scale that necessitates Marshall’s notion of input sharing. We will refer to this channel as *cost reduction through competition.*

The second channel though which hospital concentration leads to reduced input costs is the traditional Marshallian input sharing mechanism. In the Marshallian framework, input production of medical services involves scale economies. Concentrations of downstream hospitals form a large potential outsourcing demand for intermediate inputs, which allows specialized input providers to achieve an efficient scale of production and thereby provide the input services at a lower cost. We will refer to this channel as *cost reduction through specialization.*

Both mechanisms generate similar predictions: a higher concentration of medical services increases the scale of the intermediate medical industry and the increased scale of the intermediate industry then results in reduced cost of producing medical inputs. Also predicted by both models is that the cost of entry does not affect the price of lab tests directly and serves as an ideal instrument for the size of the local medical laboratory.^8^

### Cost reduction through competition

To illustrate the first cost reduction channel though competition, we adopt the model proposed by Ono [6], which assumes that final producers purchase all intermediate services locally. This is likely to be true for the health care industry when hospitals send physical samples to be tested by nearby specialized medical labs.^9^ For simplicity, the model assumes that only one intermediate input is needed for production.

The model is completed by solving for the equilibrium market price of the intermediate service and the equilibrium number of service producers. To achieve this goal, we first analyze how intermediate service producers set the price and the quantity of the service provided by maximizing their profits. This depends on both the market demand for intermediate medical services and the cost function associated with providing these services. The full derivation of the model can be found in the Additional file 1.

The key implication of the model is that decision on whether to enter the market or not is based on anticipated profit and the cost of entry. We assume that a service supplier will enter the market if its anticipated profit is at least the same size as the cost of entry. The model then gives us,

1 $$ \left(P-\varpi \right)\frac{Q}{M}=C, $$

where *P* is the market price of the service, *C* represents the cost of entry, *ϖ* is the cost of production of the intermediate service, *Q* is the outsourcing demand and *M* is the number of input producers. This suggests that the equilibrium number of service suppliers, M*, will be a function of two exogenous factors that enter the system, M* (C, *N*). This conclusion also implies that the cost of entry for intermediate service suppliers, *C*, has a direct impact on the equilibrium number of service suppliers (M*) but not on the equilibrium quantity or the price of the inputs provided (P*, Q*). Its impact on the price of intermediate services is solely through its effect on *M* since P* = *p* [M* (C, *N*), *N*]. This leads to an ideal instrument (i.e., cost of entry) for the size of the intermediate industry when attempting to identify the extent to which the entry of medical labs results in a reduced price of intermediate medical services.

### Cost reduction through specialization

The model for cost reduction through specialization departs from the model for the competition mechanism by assuming that there is only one intermediate service supplier. The supplier decides whether or not to enter the market and if so decides on its optimal level of production. This firm gains from returns to scale, which provides benefits to production as the intermediate supplier grows. The full derivation of this model can be found in the Additional file 1. The result of the model is once again that the cost of entry impacts the size of the intermediate industry, and that the price of the intermediate service is a function of the industry size. This result, which is the same for both the competition case and the specialization case, is summarized in Proposition 1.

***Proposition 1:*** *The cost of entry for intermediate medical service suppliers has a direct impact on the number of service suppliers (competition case) or the amount of lab employment (specialization case) in the local market. It affects the equilibrium price of intermediate services only through its impact on the size of the local intermediate input industry.*

### Comparative statics

Of interest is how a change in concentration of the hospital service industry affects the likelihood that independent medical labs choose to locate in the same area or how big the local medical lab industry is likely to be; as well as how increased intermediate input services in locations with concentrated services lead to lower intermediate input prices. To this end, both models are solved for comparative statics, which are summarized in Proposition 2. Both models suggest that the size of the intermediate industry increases with the degree of the hospital concentration. Further, the presence of a sizable intermediate medical industry reduces the cost associated with obtaining medical laboratory tests. This hypothesis will be empirically tested in the following context.

***Proposition 2:*** $\frac{\partial {M}^{*}}{\partial N}>0$ *and* $\frac{\partial {P}^{*}}{\partial N}<0$ *if the marginal revenue facing supplier j is steeper than the demand function (competition case) or the marginal revenue facing the single input supplier is steeper than the marginal cost function which is downward sloping (specialization case).*

(Proof of Proposition 2 is provided in the Additional file 1)

## Data

The data for our analysis come from several different sources. Price data come from the Medical/Surgical Module of the FAIR Health database between 2003 and 2010.^10^ The FAIR Health data is a collection of claims submitted from providers to private insurance companies. Though submission to the FAIR Health data is voluntary, there is evidence that the data are nationally representative [7]. Within a claim, individual procedures and their specific prices can be identified by line item using the American Medical Association’s Current Procedure Terminology (CPT) codes. For a given line item, we observe the date for which the procedure was billed, the three-digit zip code, and the amount that the insurance company reimbursed the service provider, known as the allowed amount, and any modifier codes.^11^

The reimbursement amount is not a direct measure of the cost of the intermediate service. It is a function of the bargaining process between insurance companies and hospitals. There is however reason to believe that despite the noise introduced by the bargaining process, the reimbursement amount is still highly correlated with the true cost of a procedure. Clemens and Gottlieb [8] demonstrate that private payments are heavily influenced by prices set by Medicare, which are in turn a direct function of Medicare’s cost estimates for procedures.

Since there are as many as several hundred intermediate medical procedures in the data as identified by their unique CPT codes, we will select a small subset of the procedures for the purpose of this study. Selection of the interested intermediate medical procedures is based on the frequency that a service has been identified as outsourced using modifier code 90. Specifically, we focus on laboratory tests with the top ten most frequent occurrences of modifier code 90.^12^ This provides us with an objective way of selecting the procedures to study. Table 1 lists the 10 laboratory tests with the most frequent incidence of modifier code 90 in the data. Descriptions of each procedure can be found in the Additional file 1.^13^ The price associated with each intermediate medical procedure is aggregated at the three-digit zip code level – the smallest geographic identifier available in the FAIR Health data. The summary statistics for the average price of each procedure focused on in this study are reported in Table 2.

**Table 1:** Laboratory tests with the most frequent modifier code 90 occurrences

Rank	CPT code	Description
1	80048	Basic Metabolic Panel
2	80050	General Health Panel
3	80051	Electrolyte Panel
4	80061	Lipid Panel
5	80076	Hepatic Function Panel
6	81001	Urinalysis with Microscopy
7	81003	Urinalysis without Microscopy
8	82043	Urine Test for Renal Disease or Diabetes
9	82105	Test for Alpha-Fetoprotein Levels
10	82150	Another Hepatic Function Panel

**Table 2:** Summary statistics

	Mean	Std. dev.
Price of		
Basic Metabolic Panel (CPT 80048)	10.43	7.13
General Health Panel (CPT 80050)	33.50	22.03
Electrolyte Panel (CPT 80051)	8.41	7.79
Lipid Panel (CPT 80061)	16.88	10.47
Hepatic Function Panel (CPT 80076)	11.12	7.71
Urinalysis with Microscopy (CPT 81001)	5.19	8.33
Urinalysis without Microscopy (CPT 81003)	3.62	3.02
Urine Test for Renal Disease or Diabetes (CPT 82043)	10.29	8.08
Test for Alpha-Fetoprotein Levels (CPT 82105)	19.93	16.51
Another Hepatic Function Panel (CPT 82150)	8.13	6.24
Total Number of Hospital Beds (100 s)	11.32	12.96
Medical Lab Employment (100 s)	3.49	65.56
Population Density (1,000 s per Square Mile)	0.70	3.40
Area (1,000 s of Square Miles)	3.79	4.41
Average Size of Local Hospitals (100 s of Beds)	1.51	0.92
Lab Technician Licensure Requirements	0.2222	0.4157
Percentage of Uninsured Population	0.1976	0.0570
Per Capita Income (USD)	34757.31	10742.11
Percentage of Lower than High School Education	0.0784	0.0424
Percentage of Non-White	0.1625	0.1306
Percentage of Greater than 65 Years Old	0.1403	0.0297

To help explain the price variation in intermediate medical services across different locations, we obtain data on the size of the local hospital industry, the amount of medical lab employment, the size of the local area, local population counts, as well as other geographic and demographic attributes from a variety of data sources. The number of hospital beds, which serves as a measure for the agglomeration extent of the local medical industry (especially once population density is controlled for), is obtained from the 2009 Provider of Services (POS) File. The POS file is collected by the Centers for Medicaid and Medicare Services and contains information, such as the number of hospitals and the available hospital beds, at the five-digit zip code level.^14^

Medical lab employment data are from the Dun and Bradstreet (D&B) Marketplace data files for the third quarter of 2007. These data are collected by Dunn and Bradstreet, a for-profit firm, and contain a wealth of information on businesses. This includes detailed information on the industry to which each business establishment in the data belongs, the number of workers onsite, as well as the five-digit zip code where the business is located. The industry information is identified by six-digit North American Industry Classification System (NAICS) codes. We use information on businesses with NAICS code 621511, which identifies independent medical laboratories.

Data on social economic attributes are obtained from the 2010 Area Resource File (ARF), which is published by the Health Resources and Services Administration. This file provides a set of social economic attributes at the county level, of which we include the following as further controls: the percentage of the population without health insurance, the percentage of the population greater than 65 years old, per capita income, the percentage of the population which is Non-White, and the percentage of the population with lower than a high school education. The final data source is the U.S. census from which we obtain information on area size and population density.

The various data sources were merged together via three-digit zip codes. Most data sources used (The Fair Health data, the D&B data file, and the US Census) provide information at the five-digit zip code level, making data merges relatively simple. The ARF data is provided at the county level. In this case data were matched by an area weighted average of the variable of interest based on how much land area the two geographic units in question (the county area and the three-digit zip code area) have in common. Summary statistics for these variables are presented in Table 2.

The information on medical lab technician licensing requirements comes from the American Society for Clinical Pathology (ASCP). The ASCP promotes personnel standards that include certification, licensure, and practice requirements. Eleven states out of fifty license laboratory personnel as of November 2009: California, Florida, Hawaii, Louisiana, Montana, Nevada, New York, North Dakota, Rhode Island, Tennessee, and West Virginia. Of these states, New York, North Dakota, and California enacted their licensing requirements during the time frame of our sample (New York in 2006, North Dakota in 2006, and California in 2007), although for the purposes of identification we will be using the geographic differences in the laws rather than the temporal differences as our measure of lab employment is only observed in one year. For the purpose of this study, we code the licensing requirements equal to one if a state has a licensing law active for its in-state medical laboratory personnel in a specific year.

Data were matched into the FAIR Health data by year when possible. This was done for all data other than the Dunn and Bradstreet data or the number of hospital beds, as they were only available for a single year. These data were matched such that the single year of data populated the same field for all years in the FAIR Health data.

It is important to note that because we are matching data from different years into a single analysis file, and as we are using a single year of hospital bed data, we are implicitly assuming that the density of hospital beds is relatively stable over time. If the number of beds changes rapidly then it would be invalid to estimate prices in 2006 or 2007 based on the number of beds in a geographic area in 2009. Fortunately, this is not the case. The Dartmouth Atlas tracks the number of acute care hospital beds, and reports the number of beds per resident in 2006 and 2012. Over this time frame in the 200 most populated hospital referral regions the average change in the number of beds per 1,000 residents was -0.36 beds. Only 5 out of 200 of the regions had a change with a magnitude greater than or equal to 1 bed per 1,000 residents, with the largest change being -1.1 beds per 1,000 residents. Overall this is a very slow rate of change, lending validity to the choice of using one year of hospital bed data to represent hospital agglomeration for several years of data.

## Methods

All analysis conducted in this study used STATA statistical software. The first question that we explore is whether agglomeration economies facilitate cost savings for laboratory tests. To this end, we begin by estimating a model with the following specification via ordinary least squares (OLS),

2 $$ {P}_{it}={\beta}_0+{\beta}_1 Bed{s}_i+{\beta}_2 PopDe{n}_i+{\beta}_3 Are{a}_i+{\beta}_4 AveSiz{e}_i+{\beta}_5{X}_i+ Tren{d}_t+ Regio{n}_i+ Tren{d}_t\times Regio{n}_i+{\varepsilon}_{it} $$

where, *P*_*it*_ is the average price for a medical laboratory test in three digit zip code area *i* in year *t*; *Beds*_*i*_ is the number of hospital beds in area *i* (does not very over time); *PopDen*_*i*_ represents population density in area *i*; and *Area*_*i*_ is the area of the three digit zipcode *i* in square miles. Controlling for these two variables is important for three reasons. First, holding population constant is important for measuring how concentrated the hospital industry is – there is a large difference between 500 beds for a population of 5,000 and 500 beds for a population of 50,000. Second, demand for health services is largely dependent on population. Many health services are consumed only when an adverse health event occurs. These events occur with some probability for each individual, making health services subject to a demand function that is due in large part to the size of the local population. Third, prices vary with the degree to which an area is developed. The same procedure may be charged for a higher price in more densely populated areas. All these considerations make controlling for population density important when estimating the price of health services. Once population density is controlled for, *β*_1_ gives an estimate of the effect of hospital services density on the price of the intermediate input.

Another factor that is important in influencing the price of intermediate medial services is the size composition of local hospitals. On the one hand, large hospitals may be more proficient in exploring internal economies of scale, which are likely to reduce the cost of producing intermediate medical services in-house. On the other hand, small hospitals may be more beneficial in creating a community of independent suppliers [9] which tends to enhance the input sharing mechanism and reduce the price of intermediate services.^15^ Which one of the two effects dominates is unclear. We include *AveSize*_*i*_, the average size of the local hospitals, which is assumed not to vary over time, to capture such an effect. *X*_*i*_ is a vector of controls which includes the percent of the local population that are uninsured, the percent that are unemployed, the percent with less than a high school education, the percent that are non-white, the percent that are over 65 years of age, and the median household income. *Trend*_*t*_ is a vector of year trends to capture nationwide price inflation. This variable is further interacted with a region dummy to capture any region-specific year trends in inflation. These variables also capture any region level unobservable omitted variables that either do not vary with time or that vary over time in a linear fashion.

Next we explore whether the impact of agglomeration economies on input prices arises from the ability to share intermediate input providers. First we examine whether an increase in hospital density is associated with an increase in the size of independent laboratory employment. We do so by estimating the following specification via OLS, in which we change the dependent variable in Eq. (2) to the size of the local intermediate medical industry as measured by independent medical laboratory employment in 2007,

3 $$ Lab{E}_i={\beta}_0+{\beta}_1 Bed{s}_i+{\beta}_2 PopDe{n}_i+{\beta}_3 Are{a}_i+{\beta}_4 AveSiz{e}_i+{\beta}_5{X}_i+ Regio{n}_i+{\varepsilon}_i $$

Then, to get a better sense of how the reduced price of medical laboratory tests is due to the increase in the medical lab employment, we re-estimate Eq. (2) after including *LabE*_*i*_ as an additional explanatory variable,

4 $$ {P}_{it}={\beta}_0+{\beta}_1 Bed{s}_i+{\beta}_2 Lab{E}_i+{\beta}_3 PopDe{n}_i+{\beta}_4 Are{a}_i + {\beta}_5 AveSiz{e}_i+{\beta}_6{X}_i+ Tren{d}_t+ Regio{n}_i + Tren{d}_t\times Regio{n}_i+{\varepsilon}_{it} $$

This specification provides a general association between lab employment and the price of laboratory tests. However, as medical lab employment is simultaneously determined with the price of the intermediate medical services and higher prices may attract more medical labs to the local area, the estimated *β*_2_ in this case will be biased due to endogeneity arising from reverse causality.

To deal with this issue we re-estimate Eq. (4) with two stage least squares (2SLS), using variation in state lab technician licensure laws as an instrument for medical laboratory employment. Specifically, state licensure laws create spatial variation in the cost of hiring lab workers, which in turn creates clean variation in how agglomeration can translate into growth in the intermediate industry. Though licensure laws do not fit the criteria of an ideal instrument as proposed by the theoretical model, they do pass the exclusion restriction needed for identification in a reduced form setting. Identification is based on state licensure laws only impacting the cost of intermediate services through the cost of running a medical lab. The laws have no direct effect on the price of intermediate tests, are not likely reversely determined by the price of tests, and do not impact their price through any channel other than the cost of intermediate input production. The 2SLS estimation provides a refined window into the mechanism of agglomeration economies. In the first stage we are able to see how hospital density impacts the size of the intermediate input industry. Then in the second stage we can observe how the size of the intermediate industry affects the price of the intermediate input directly.

## Results

### Agglomeration’s impact on input prices

Tables 3 and 4 report results for two of the laboratory tests focused on in this study: Table 3 contains results for a basic metabolic panel (CPT 80048), and Table 4 reports results for a urinalysis with microscopy (CPT 81001). Results from the OLS estimations are reported in columns (1) through column (3) of both tables. The first column reports estimates for Eq. (2), the second column reports estimates for a regression using lab employment as the dependent variable (Eq. (3), and the third column reports the OLS estimates for Eq. (4). The 2SLS estimates are reported in column (4) and column (5) of both tables.

**Table 3:** Basic Metabolic Panel (80048, Blood Test) (t stats are reported in parentheses using robust standard errors)

Model	OLS	OLS	OLS	2SLS	
				1^st^ stage	2^nd^ stage
*Dependent variable*	Average price	Lab employment	Average price	Lab employment	Average price
	(1)	(2)	(3)	(4)	(5)
*Independent variables*					
Total Number of Hospital Beds (100 s)	−0.048***	32.085***	−0.028***	32.162***	0.681***
	(-7.15)	(25.77)	(-3.82)	(25.69)	(2.86)
Medical Lab Employment (100 s)	-	-	−0.0632***	-	−2.273***
	-	-	(-5.44)	-	(-3.05)
Population Density (1,000 s per Square Mile)	0.229***	7.622*	0.234***	7.859*	0.403***
	(4.89)	(1.69)	(5.02)	(1.75)	(3.40)
Area (1,000 s of Square Miles)	0.257***	−18.468***	0.245***	−18.063***	−0.165
	(8.00)	(-13.09)	(7.61)	(-12.49)	(-1.13)
Average Hospital Size (100 s of Beds)	−0.419***	−63.206***	−0.459***	−60.800***	−1.864***
	(-3.65)	(-6.20)	(-3.98)	(-5.81)	(-3.19)
Lab Technician Licensing Laws	-	-	-	−60.772***	-
	-	-	-	(-3.39)	-
Demographic Controls^a^	YES	YES	YES	YES	YES
Year Trend	YES	NO	YES	YES	YES
Region Fixed Effect	YES	YES	YES	YES	YES
Year Trend × Region Fixed Effect	YES	NO	YES	YES	YES
No. of observations	6334	6344	6334	6334	6334
R-squared	0.209	0.515	0.210	0.515	-
Root MSE	6.379	456.31	6.373	456.31	11.88
First-stage F-statistics	-	-	-	11.508	
Wooldridge Robust Score Test	-	-	-	33.874	

^a^Other control variables include county-level measure of % of uninsured, % of > 65 years old, per capita income, % Non-white, and % of < high school. Statistical significance at the 1, 5 and 10 percent levels are denoted with ***, **, and * respectively

**Table 4:** Urinalysis with Microscopy (81001, Urine Test) (t stats are reported in parentheses using robust standard errors)

Model	OLS	OLS	OLS	2SLS	
				1^st^ stage	2^nd^ stage
*Dependent variable*	Average price	Lab employment	Average price	Lab employment	Average price
	(1)	(2)	(3)	(4)	(5)
*Independent variables*					
Total Number of Hospital Beds (100 s)	−0.017***	32.114***	−0.009	32.204***	0.122
	(-3.07)	(25.74)	(-1.38)	(25.66)	(1.64)
Medical Lab Employment (100 s)	-	-	−0.024***	-	−0.432*
	-	-	(-3.37)	-	(1.88)
Population Density (1,000 s per Square Mile)	0.045	7.659*	0.047	7.942*	0.078*
	(1.38)	(1.70)	(1.44)	(1.77)	(1.88)
Area (1,000 s of Square Miles)	0.075***	−18.726***	0.071**	18.258***	−0.006
	(2.57)	(-13.18)	(2.46)	(-12.52)	(-0.12)
Average Hospital Size (100 s of Beds)	−0.294**	−63.089***	−0.309**	−6.050***	−0.568***
	(-2.03)	(-6.18)	(-2.16)	(-5.79)	(-2.68)
Lab Technician Licensing Laws	-	-	-	−63.36***	-
	-	-	-	(-3.53)	-
Demographic Controls^a^	YES	YES	YES	YES	YES
Year Trend	YES	NO	YES	YES	YES
Region Fixed Effect	YES	YES	YES	YES	YES
Year Trend × Region Fixed Effect	YES	NO	YES	YES	YES
No. of Observations	6309	6326	6309	6309	6309
R-squared	0.038	0.518	0.039	0.514	-
Root MSE	8.212	457.170	8.212	457.231	8.379
First-stage F-statistics	-	-	-	12.524	
Wooldridge Robust Score Test	-	-	-	4.095	

^a^Other control variables include county-level measure of % of uninsured, % of > 65 years old, per capita income, % Non-white, and % of < high school. Statistical significance at the 1, 5 and 10 percent levels are denoted with ***, **, and * respectively

The estimates in column (1) of both tables suggest that an increase in the scale of the hospital service industry decreases the price of medical laboratory tests. This is obtained without controlling for the local medical lab employment. The magnitude of the impact is 0.048 dollars for Basic Metabolic Panel and 0.017 dollars for Urinalysis with Microscopy, for an increase of 100 hospital beds in a three-digit zip code area. The regression also produces a positive coefficient for population density, a positive coefficient for the physical size of the area, and a negative coefficient for the average size of the local hospitals all of which are significant at conventional levels. These estimates have intuitive interpretations. First, higher demand as captured by larger population density increases the price of intermediate medical services. Second, larger physical area is generally associated with higher input prices. This might be because the larger an area is physically, the higher the average transportation cost is. This likely increases the cost of outsourcing, which increases the price of the lab tests. Third, average hospital size in the area reflects, from one perspective, the extent to which large hospitals are capable of exploiting internal economies of scale and, from another perspective, how groups of small hospitals attract intermediate suppliers. The former suggests that a higher average size of local hospitals will reduce the cost of producing lab tests, while the latter tends to predict a positive sign. The negative coefficient associated with this variable suggests that it is the internal economies of scale mechanism that dominates this effect.

Column (2) of both Tables 3 and 4 explores the relationship between hospital concentration and the size of the intermediate industry. The estimated coefficient associated with the number of hospital beds is positive and statistically significant at conventional levels for both tables. This implies that independent medical labs tend to locate where the nearby hospital industry is of larger scale. The magnitude is estimated to be as large as a 32 lab employee increase per additional 100 hospital beds. Additionally, higher population density is also associated with more lab employment and larger physical area is associated with less medical lab employment. An increased average hospital size is associated with lower independent lab employment, as larger hospitals are more likely to be able to support an in-house lab.

Column (3) reports estimates from the same regression as column (1) with laboratory employment as an additional regressor. Larger medical lab employment is associated with a reduced price for both of the tests examined. As the same time, the estimate for the effect of additional hospital beds is smaller when the additional regressor is included. This suggests that part of the agglomeration effect measured by the number of beds is absorbed by the inclusion of medical lab employment. This is only a rough story; in order to precisely split out the agglomeration effect into its parts we need to rely on the instrument in our next analysis. The estimates associated with other control variables are generally consistent with our previous findings.

The first stage of the 2SLS regression is reported in column (4) of Tables 3 and 4. Similar to what is shown in column (2) of both tables, an increase in the number of hospital beds is positively associated with a larger size for the intermediate laboratory industry. This provides evidence that increasing the density of the hospital industry in a three-digit zip code area increases the size of intermediate input suppliers. At the same time licensing laws are associated with statistically significant decreases in intermediate lab employment. Specifically, the adoption of state licensing laws reduces independent lab employment by approximately 61 employees. F-statistics in the first stage are above 10 in both cases.

The second-stage 2SLS estimates are reported in column (5) of Tables 3 and 4. Once laboratory employment is instrumented for, there is a much stronger negative effect of outside laboratory employment on lab test prices. For instance, an increase in lab employment of 100 employees is associated with a $2.27 decrease in the cost of the Basic Metabolic Panel. Columns (4) and (5) together provide evidence that increased hospital density increases the size of the intermediate industry (which is observed in the first stage), which in turn lowers the cost of intermediate inputs (which is observed in the second stage), generating an agglomeration benefit. The magnitude of the impact is larger than the OLS estimates (-2.273 from the 2SLS regression compared to -0.063 from the OLS regression). This underlines the importance of the previously mentioned concern of bias caused by potential reverse causality. It is also important to note that the direct effect of hospital density on the price of the intermediate laboratory test is now positive and in some cases not statistically significant at conventional levels. This seems to suggest that the majority of the impact of agglomeration economies on input prices is through input sharing. Once the proxy for input sharing is controlled for, the agglomeration measure itself no longer has significant effect of reducing the input prices.

Tables 5 and 6 report estimates from the second stage of the 2SLS estimation for the remaining eight laboratory tests.^16^ All of the other tests show results similar to those for the basic metabolic panel and urinalysis with microscopy. Magnitudes range from approximately a 0.52 dollar decrease in the price of a urinalysis without microscopy per 100 additional lab employees, to approximately a 2.66 dollar decrease in the price of a lipid panel per 100 additional lab employees.

**Table 5:** The impact of agglomeration economies on various hospital input prices (t stats are reported in parentheses using robust standard errors)

Type of intermediate medical service (CPT code)	General health panel (80050)	Electrolyte panel (80051)	Lipid panel (80061)	Hepatic function panel (80076)
	(1)	(2)	(3)	(4)
Total Number of Hospital Beds (100 s)	3.179***	0.541***	0.804***	0.567***
	(3.22)	(2.75)	(2.86)	(2.75)
Medical Lab Employment (100 s)	−1.078***	−1.830***	−2.663***	−1.900***
	(-3.26)	(-2.97)	(-3.02)	(-2.95)
Population Density (1,000 s per Square Mile)	0.621	0.275**	0.434***	0.290***
	(1.21)	(2.43)	(3.14)	(2.70)
Area (1,000 s of Square Miles)	−1.173*	−0.139	−0.191	−0.075
	(-1.93)	(-1.12)	(-1.11)	(-0.57)
Average Hospital Size (100 s of Beds)	−6.315***	−1.273**	−2.104***	−1.446***
	(-2.58)	(-2.44)	(-3.06)	(-2.87)
Demographic Controls^a^	YES	YES	YES	YES
Year Trend	YES	YES	YES	YES
Region FE	YES	YES	YES	YES
Year Trend × Region FE	YES	YES	YES	YES
No. of Observations	6297	5873	6373	6304
Root MSE	49.981	11.043	15.063	10.801
First-stage F-statistics	12.474	13.471	12.270	11.973
Wooldridge Robust Score Test	65.901	20.759	25.741	22.299

^a^Other control variables include county-level measure of % of uninsured, % of > 65 years old, per capita income, % Non-white, and % of < high school. Statistical significance at the 1, 5 and 10 percent levels are denoted with ***, **, and * respectively

**Table 6:** The impact of agglomeration economies on various hospital input prices (t stats are reported in parentheses using robust standard errors)

Type of intermediate medical service (CPT code)	Urinalysis without microscopy (81003)	Urine test for early renal disease or diabetes (82043)	Test for alpha-fetoprotein levels (82105)	Another hepatic function panel (82150)
	(1)	(2)	(3)	(4)
Total Number of Hospital Beds (100 s)	0.155**	0.507***	0.469	0.316**
	(2.55)	(2.63)	(1.60)	(2.22)
Medical Lab Employment (100 s)	−0.524***	−1.667***	−1.633*	−1.100***
	(-2.75)	(-2.76)	(-1.78)	(-2.47)
Population Density (1,000 s per Square Mile)	0.090***	0.134	0.244	0.221***
	(3.06)	(1.41)	(1.60)	(3.22)
Area (1,000 s of Square Miles)	−0.020	−0.052	0.271	−0.059
	(-0.53)	(-0.43)	(-1.43)	(-0.66)
Average Hospital Size (100 s of Beds)	−0.403***	−1.291***	0.980	−1.315***
	(-2.62)	(-2.70)	(-1.40)	(-3.72)
Demographic Controls^a^	YES	YES	YES	YES
Year Trend	YES	YES	YES	YES
Region FE	YES	YES	YES	YES
Year Trend × Region FE	YES	YES	YES	YES
No. of Observations	6332	6159	5509	6146
Root MSE	3.640	11.032	7.315	7.406
First-stage F-statistics	13.017	12.345	13.452	11.190
Wooldridge Robust Score Test	14.920	15.643	3.603	11.183

^a^Other control variables include county-level measure of % of uninsured, % of > 65 years old, per capita income, % Non-white, and % of < high school. Statistical significance at the 1, 5 and 10 percent levels are denoted with ***, **, and * respectively

### Competition or specialization? Some suggestive evidence

One remaining question about our results is whether the cost reductions observed occur because of cost reduction through competition or because of cost reduction through specialization. We provide some suggestive evidence by estimating an equation qualitatively similar to Eqn (3). We first replace the dependent variable of the amount of laboratory employment with the number of labs total, which we obtained from the U.S. Census, and then we stratify by the amount of employment in the labs. If increasing hospital density increases the number of smaller labs, then that could be seen as evidence in favor of cost reduction through competition. On the other hand, if increasing hospital density increases the number of large labs, then that would be less indicative of cost reduction through competition.

Results of this analysis are presented in Table 7. The only statistically significant effect of hospital density on the number of labs occurs for medical labs with a total employment of 500 or more employees, an effect of an additional 2.74 labs per 100,000 hospital beds. This result is suggestive that scale economies within a lab, or specialization, is a major driving force in cost reductions for intermediate inputs. However, this does not completely rule out cost reduction though competition. Even though the additional labs that opened due to hospital density were quite large, they were still additional labs, and as such did push the market for intermediate services towards competitiveness.

**Table 7:** The impact of agglomeration economies on the number of medical labs of different sizes (t stats are reported in parentheses)

Medical labs of different sizes	Medical Labs with 1-19 employment	Medical Labs with 20-99 employment	Medical Labs with 100-499 employment	Medical Labs with 500 or more employment
	(1)	(2)	(3)	(4)
Total Number of Hospital Beds (100,000 s)	0.413	0.304	−0.0718	2.740***
	(1.29)	(0.93)	(-0.14)	(4.41)
Population Density (100,000 s per Square Mile)	0.219	−0.013	0.858***	−1.181***
	(1.36)	(-0.08)	(3.24)	(-3.77)
Area (100,000 s of Square Miles)	−0.212*	0.875***	0.534***	−1.873***
	(-1.96)	(7.89)	(-1.43)	(-8.90)
Average Hospital Size (100 s of Beds)	−0.010	−0.042***	−0.168***	0.233***
	(-1.46)	(-5.88)	(-14.69)	(17.34)
Demographic Controls^a^	YES	YES	YES	YES
No. of observations	4661	4661	4661	4661
R-Squared	0.004	0.047	0.151	0.233

^a^Other control variables include county-level measure of % of uninsured, % of > 65 years old, per capita income, % Non-white, and % of < high school. Statistical significance at the 1, 5 and 10 percent levels are denoted with ***, **, and * respectively

### Laboratory employment or laboratory wages? Further suggestive evidence

One question about our results is whether our measure of laboratory employment is truly capturing the size of the intermediate industry. It is possible that laboratory employment is simply serving as a proxy for the intermediate industry labor market equilibrium in general, and that what we are measuring is an amalgam of both employment and wage effects. To address this possibility we run our analysis using a measure of the wages of laboratory workers obtained from the American Community Survey. The average weekly wage is $1169.25 with a standard deviation of $646.41.

The results of this analysis are presented in Table 8 for a basic metabolic panel and in Table 9 for a urinalysis with microscopy (the results are presented in the same format as Tables 3 and 4b which look at the same tests). Unlike laboratory employment, the wage is not responsive to the density of beds in a geographic area at standard levels of statistical significance.^17^ Further, there appears to be little if any direct effect of the wage on the price of laboratory tests. In the case of Blood Tests, we do see that the price has been pushed up slightly due to higher wage level (Table 8). In the case of Urine Tests, the estimate is insignificant at conventional levels (Table 9). Since the general impact, if it exists, goes in the opposite direction of our main estimates, Tables 8 and 9 show that the magnitude identified in Tables 3 and 4 can be interpreted as a lower bound. To the extent that still find a significant negative impact of agglomeration on input prices, even though higher wages may push up the price, it is sensible to conclude that agglomeration economies have some negative impact on hospital input prices. These results suggest that to the extent our results are driven by the labor market for laboratory technicians it is mainly through the increase in employment and with it the increase in the size of the intermediate industry.

**Table 8:** Effect of wages for basic metabolic panel (80048, blood tests) (t stats are reported in parentheses using robust standard errors)

Model	OLS	OLS	2SLS	
			1^st^ stage	2^nd^ stage
*Dependent variable*	Lab wages	Average price	Lab wages	Average price
	(2)	(3)	(4)	(5)
*Independent variables*				
Total Number of Hospital Beds (100 s)	−0.007	0.0386***	−1.743	0.041***
	(-0.57)	(3.54)	(-1.50)	(3.56)
Medical Lab Wage	-	−0.000	-	0.004**
	-	(-.038)	-	(5.89)
Population Density (1,000 s per Square Mile)	0.202***	−0.003***	0.208***	−0.004***
	(4.32)	(-8.46)	(4.69)	(-9.23)
Area (1,000 s of Square Miles)	−7.741***	0.310***	−10.112***	0.338***
	(-5.06)	(8.78)	(6.45)	(9.29)
Lab Technician Licensing Laws	-	-	446.104***	-
	-	-	(19.24)	-
Demographic Controls^a^	YES	YES	YES	YES
Year Trend	YES	YES	YES	YES
Region Fixed Effect	YES	YES	YES	YES
Year Trend × Region Fixed Effect	YES	YES	YES	YES
No. of Observations	5122	5122	5122	5122
R-squared	0.194	0.194	0.245	-
Root MSE	581.95	6.586	563.719	6.864
First-stage F-statistics	-	-	69.03	
Wooldridge Robust Score Test	-	-	33.099	

^a^Other control variables include county-level measure of % of uninsured, % of > 65 years old, per capita income, % Non-white, and % of < high school. Statistical significance at the 1, 5 and 10 percent levels are denoted with ***, **, and * respectively

**Table 9:** Effect of wages for urinalysis with microscopy (81001, urine test) (t stats are reported in parentheses using robust standard errors)

Model	OLS	OLS	2SLS	
			1^st^ stage	2^nd^ stage
*Dependent variable*	Lab wages	Average price	Lab wages	Average price
	(2)	(3)	(4)	(5)
*Independent variables*				
Total Number of Hospital Beds (100 s)	−0.662	0.029***	−0.017	0.029***
	(-0.54)	(4.15)	(-1.46)	(4.21)
Medical Lab Wage	-	0.000	-	0.001
	-	(0.52)	-	(1.55)
Population Density (1,000 s per Square Mile)	0.202***	−0.002***	0.210***	−0.002***
	(4.32)	(-5.71)	(4.74)	(4.21)
Area (1,000 s of Square Miles)	−8.050***	0.110***	−10.674***	0.114***
	(-5.24)	(4.62)	(-6.81)	(4.82)
Lab Technician Licensing Laws	-	-	445.607***	-
	-	-	(19.12)	-
Demographic Controls^a^	YES	YES	YES	YES
Year Trend	YES	YES	YES	YES
Region Fixed Effect	YES	YES	YES	YES
Year Trend × Region Fixed Effect	YES	YES	YES	YES
No. of Observations	5097	5097	5097	5097
R-squared	0.195	0.036	0.245	-
Root MSE	582.33	9.014	564.691	8.960
First-stage F-statistics	-	-	68.30	
Wooldridge Robust Score Test	-	-	1.648	

^a^Other control variables include county-level measure of % of uninsured, % of > 65 years old, per capita income, % Non-white, and % of < high school. Statistical significance at the 1, 5 and 10 percent levels are denoted with ***, **, and * respectively

## Discussion

It is a useful exercise to put the results of this study into context in terms of magnitude. We find that increasing hospital bed density by 100 beds in a geographic area is associated with an additional 32 lab employees, or an increase of approximately 9 percent off of the average. An additional 100 employees is associated with lower lipid panel prices of $2.66 or approximately a 16.8 percent decrease off of the average. So, if we set all variables to their population averages, an increase in beds of approximately 10 percent (or about 110 beds) would be associated with a downstream decrease in lipid panel prices of $0.94 or a cost decrease of 5.6 percent off of the average. This is roughly half the size of the effect found by Cohen and Paul [2] for an increase of 1 % in employment or proximity to expertise in other hospitals, and a little over a third of the cost saving from hospital consolidation found by Dranove and Lindrooth [10].

## Conclusion

This study examines whether agglomerated hospital services are important in generating spillover effects, which reduces the cost of producing health care. Among various channels through which agglomeration economies might take place in the hospital service industry, we focus specifically on intermediate input sharing. Higher demand for intermediate inputs from concentrated hospital services increases the tendency of specialized intermediate medical labs to locate at these places. The increased presence of medical laboratories then reduces the price of intermediate medical services at concentrated locations due to competition or specialization, or possibly both.

Our analysis shows that the size of the hospital service industry has a strong impact on reducing the price of intermediate medical inputs. Further, the reduced price at concentrated locations is mainly caused by the large presence of the intermediate medical laboratory industry. This evidence provides another important piece in the growing body of evidence as to the source, size and nature of agglomeration economies.

The results also yield strong policy implications for the health care sector. Cost savings in the provision of medical care have become increasingly important in light of the enormous growth in medical spending and the subsequent strain that it has put on both family and public budgets. When determining where to locate practices and hospitals, or when deciding which existing hospitals to expand, cost savings from input sharing, as well as other agglomeration sources should certainly be taken into consideration.

## Additional file

Additional file 1: Supplementary Materials.(DOCX 38 kb)

## Footnotes

1
http://www.dartmouthatlas.org/works

2 Previous studies examining the impact of health care capacity find inconsistent evidence. Studies using available cross-sectional datasets show almost complete absence of a positive relationship between medical investment and the quality of care ([11, 12]; [13]; [14]). In contrast, studies based on instrumental variables, panel data, or other differencing strategies suggest that higher medical investment is associated with significantly lower mortality ([15]; [16]; [17]; [18]; [19]; [20]).

3 Numerous studies have provided evidence that external economies of scale enhance productivity in the manufacturing sector, such as Nakamura [21], Henderson [22]; Ellison and Glaeser [23], Duranton and Puga [24], Ellison et al. [25], Behrens and Bougna [26], etc. A few studies have focused on the service sector [27, 28]. Bates and Santerre [1], Baicker and Chandra [29], Cohen and Paul [2], and Li [4] are among the few that examine productivity gains from agglomeration in the health care industry.

4 Agglomeration economies were first recognized by Marshall [30] in his classic textbook. This concept has been surveyed extensively in the later literature [31-35].

5 For instance, Glaeser and Maré [36] showed that a significant fraction of the urban wage premium accrues to workers by the spread of knowledge and stays with them even when they leave cities; and Costa and Kahn [37] documented that big cities are effective in solving the colocation problem for dual career households by providing a large labor market pool. Other work on knowledge spillovers and labor market pooling include Rosenthal and Strange [38], Moretti [39], Ellison, et al. [25], etc.

6 One feature of hospital agglomeration is that hospitals not only gain benefits in the form of reduced costs, but also in the form of improved quality. For example, Baicker and Chandra [29] show that hospitals located near other hospitals which provide high quality care in a short time horizon begin to provide higher quality care themselves. By focusing on a set of standard intermediate medical laboratory tests, we implicitly assume that the quality of the tests is homogenous and that any improved productivity is hence reflected in cost reductions.

7 Lower input prices at concentrated places may arise from two different sources as laid out in detail in the supplementary materials. The first is that larger markets in big cities allow input suppliers to compete with each other, which drives down the price of intermediate inputs. This type of externality is referred to as pecuniary agglomeration economies by Brueckner [5] since it is the competition among suppliers, instead of better technology, that leads to a reduction in the cost of a hospital’s inputs. The second source relies on the existence of scale economies in input production. If the input production incurs increasing returns to scale, then large outsourcing demand in hospital concentrated areas allow specialized input producers to provide the service at a lower cost by achieving an efficient scale of production. In this sense, it is the specialization in producing medical inputs that drives down the input prices. These two different sources are modeled separately, but yield similar predictions on the relationship between agglomeration extent and intermediate input prices.

8 The model has been simplified to highlight the channels through which agglomeration economies reduce the price of intermediate medical services. To keep this focus, we did not incorporate many other aspects of the medical industry that may also affect medical input costs at the same time. This includes, for instance, the potential bargaining taken place between hospitals and insurance companies as captured by the relative size of hospitals to local insurers.

9 This is less likely to be true for some other services, such as CAT scan reading, where scanned images can be easily transmitted for interpretation. This is also less likely to be true for biopsies testing for rare diseases, or for genetic tests, both of which are usually sent to one of a few national expert labs. We do not consider such services in this study.

10 FAIR Health, Inc. is an independent, New York nonprofit corporation. A thorough description of the FAIR Health database can be found in Nicholson [40].

11 When a provider submits a claim, each procedure billed is denoted by a CPT code. In addition to the CPT code, many providers will add on modifier codes to denote specific circumstances under which the procedure was done.

12 This doesn’t mean that the procedures studied in this paper are necessarily those most commonly outsourced. The absence of modifier code 90 could mean either that the procedure was done in house, or, that the provider does not use modifiers to denote outsourced procedures. Not all providers use modifiers uniformly, and while one provider may code modifiers for outsourcing, another may not. As such it is possible that procedures other than those we identify are the most commonly outsourced procedures.

13 The procedure with the most frequent occurrence of modifier code 90 was a venipuncture or drawing of blood. In this case modifier code 90 denotes that the blood sample was sent to an outside lab, not that the action of drawing blood itself was outsourced. The next 100 procedures with the most frequent occurrence of modifier 90 were all laboratory tests.

14 Details can be found at http://www.cms.gov.

15 This “Chinitz” effect has been explored in Jacobs [41], Piore and Sabel [42], Saxenian [43], Rosenthal and Strange [44], and Glaeser et al. [45].

16 OLS and first stage regression results are consistent with those found for the other two tests and are available upon request.

17 Even though we do not find an impact of industrial density on the wage in the intermediate industry, there is evidence that wages in the agglomerated industry are impacted by the agglomeration. See for example Freedman [46], Matano and Naticchioni [47] and Figueirdo et al. [48].
