# Supplementary material for: The impact of agglomeration economies on hospital input prices
Source: Health Econ Rev. 2015 Dec 8;5:38. doi: 10.1186/s13561-015-0075-1 (PMC4671981; doi:10.1186/s13561-015-0075-1)
Supplement: Supplementary file 1 — Supplementary Materials.(DOCX 38 kb) [file 13561_2015_75_MOESM1_ESM.docx]

**Additional file 1**

1. *Derivation of Cost Reduction Through Competition Model*

On the demand side, hospitals decide whether to outsource for intermediate medical services by comparing the market price of the service, $P$, to the average cost of the in-house production of the service, $\delta$. Assume that $\delta=\delta\left( A \right)+u$, where $A$ represents hospital specific characteristics (e.g., the hospital size), and the CDF for $u$ is $F(\cdot)$. The probability of outsourcing is then determined by $1-F\left[ P-\delta\left( A \right) \right]$. ^[[1]](#footnote-1)^

Suppose that there are $N$ hospitals in the market, and that the demand for intermediate services from a single hospital is $D(P)$. The overall outsourcing demand, $Q$, for intermediate services is then represented as $ND\left( P \right)\times\left\{ 1-F\left[ P-\delta\left( A \right) \right] \right\}$. This equation also gives us the inverse demand function $P=P(\frac{Q}{N})$.

The market price of an intermediate service is determined by the intermediate service supplier’s profit maximization behavior. The production technology is assumed to exhibit constant returns to scale initially.^[[2]](#footnote-2)^ The market behavior of these specialized service suppliers is assumed to follow a two-stage entry process with Cournot oligopolistic competition. The optimization is solved using backward induction. Starting from the second stage, an intermediate service producer maximizes profits $\pi=P(\frac{Q}{N})\times Q_{j} -\varpi\times Q_{j}$, where $Q_{j}$ denotes the output of a single service supplier and $\varpi$ represents the average cost of producing these services. Assuming there are $M$ input producers on the market, the first order condition for the maximization is then as follows,

|  | $\frac{\partial\pi}{\partial Q_{j}}=P\left( \frac{Q}{N} \right)+P^{'}\left( \frac{Q}{N} \right)\times\left[ \frac{Q_{j}}{N} \right]-\varpi=0$. | (S.1) |
| --- | --- | --- |

At market equilibrium, the overall outsourcing demand for intermediate medical services equals the overall supply of the services, assuming all input providers supply the same amount of services ($Q_{j}M=Q$). Substituting this condition into the above equation solves for $Q$ (as well as for $P$) as a function of the number of service suppliers, $M$, and the size of the local hospital service industry, $N$. That is to say, $P=p\left( M,N \right)$ and $Q=q(M,N)$, where $M$ is determined in the first stage when intermediate medical service suppliers decide whether or not to enter the market.^[[3]](#footnote-3)^

The decision on whether to enter the market or not is based on anticipated profit and the cost of entry. We assume that a service supplier will enter the market if its anticipated profit is at least the same size as the cost of entry. Therefore,

|  | $\left( P-\varpi\right)\frac{Q}{M}=C,$. | (S.2) |
| --- | --- | --- |

where $C$ represents the cost of entry. This suggests that the equilibrium number of service suppliers, $M^{*}$, will be a function of two exogenous factors that enter the system, $M^{*}\left( C,N \right)$. This conclusion also implies that the cost of entry for intermediate service suppliers, $C$, has a direct impact on the equilibrium number of service suppliers ($M^{*}$) but not on the equilibrium quantity or the price of the inputs provided ($P^{*}, Q^{*}$). Its impact on the price of intermediate services is solely through its effect on $M$ since $P^{*}=p[M^{*} \left( C,N \right),N]$. This leads to an ideal instrument (i.e., cost of entry) for the size of the intermediate industry when attempting to identify the extent to which the entry of medical labs results in a reduced price of intermediate medical services.

*B. Derivation of Cost Reduction Through Specialization Model*

The model for cost reduction though specialization departs from the model for the competition mechanism by assuming that there is only one intermediate medical service supplier. The single supplier decides whether to enter the market or not and, if so yes, what would be its optimal production scale (captured by $Q$) to achieve maximum profit. The production function for medical inputs is assumed to be $Q=aM^{b}$ and the technology involved has increasing returns to scale (i.e., $b>1$). ^[[4]](#footnote-4)^ This also implies that the average cost associated with producing $Q$ amount of intermediate services decreases with the output level. That is, $\varphi=\varphi\left( Q \right)$ and $\varphi^{'}<0$.

The profit function in this case is $\pi=P\left( \frac{Q}{N} \right)\times Q -\varphi\left( Q \right)\times Q-C$, where $C$ is the cost of entry, as in the previous model. Substituting $Q=aM^{b}$ into the first order condition derived below,

|  | $\frac{\partial\pi}{\partial Q}=P\left( \frac{Q}{N} \right)+P^{'}\left( \frac{Q}{N} \right)\times\left[ \frac{Q}{N} \right]-\varphi^{'\left( Q \right)}\times Q-\varphi\left( Q \right)-C=0$. | (S.3) |
| --- | --- | --- |

$M^{*}$ is solved for as a function of $N$ and $C$. That is, $M^{*}\left( C,N \right)$. Since $P=P\left( \frac{Q}{N} \right)$ and $Q$ are determined by $M$, we have $P^{*}=p[M^{*} \left( C,N \right),N]$. This result is exactly the same as in the competition scenario, where the optimal input price is determined by the scale of the local hospital service industry and the size of the medical lab industry. More importantly, the cost of entry affects the size of the local medical lab industry (in this case a single lab), but does not impact the equilibrium input price directly.

1. *Proof of Proposition 2:*

- Cost reduction by competition:

In this case, total differentiation of Eq. (3.1) and Eq. (3.2) gives us

|  | $A_{1}dQ+A_{2}dM=A_{3}dN$. | (S.4) |
| --- | --- | --- |

and

|  | $B_{1}dQ+B_{2}dM=B_{3}dN,$. | (S.5) |
| --- | --- | --- |

where $A_{1}=\left( P^{'}+\frac{P^{''}Q}{NM}+\frac{P^{'}}{M} \right)$, $A_{2}=-\frac{P^{'}Q}{M^{2}}$, $A_{3}=\left( \frac{P^{'}Q}{N}+\frac{P^{''}Q^{2}}{N^{2}M}+\frac{P^{'}Q}{NM} \right)$, $B_{1}=\left( \frac{P^{'}Q}{N}+\frac{CM}{Q} \right)$, $B_{2}=-C$, and $B_{3}=\frac{P^{'}Q^{2}}{N^{2}}$.

Applying Cramer’s rule to (A.1) and (A.2), we obtain that

|  | $\frac{dQ}{dN}=\frac{A_{3}B_{2}-B_{3}A_{2}}{A_{1}B_{2}-B_{1}A_{2}}$. | (S.6) |
| --- | --- | --- |

and

|  | $\frac{dM}{dN}=\frac{A_{1}B_{3}-B_{1}A_{3}}{A_{1}B_{2}-B_{1}A_{2}}$. | (S.7) |
| --- | --- | --- |

Since $P^{'}<0$, it follows that $A_{2}>0$, and $B_{3}<0$. We assume that $B_{2}<0$ (the entry cost is positive).

To obtain well-behaved outcomes, we assume that the marginal revenue is steeper than the demand function (Long and Soubeyran, 2000). That is,

|  | $\frac{{2P}^{'}}{N}+\frac{P^{''}Q_{j}}{N^{2}}<\frac{P^{'}}{N}$. | (S.8) |
| --- | --- | --- |

From (A.5) and $Q_{j}M=Q$, we obtain

|  | $\frac{P^{'}}{N}+\frac{P^{''}Q}{N^{2}M}<0$. | (S.9) |
| --- | --- | --- |

And it then follows from (A.6) that

|  | $A_{1}B_{2}-B_{1}A_{2}={-P}^{'}C-\frac{P^{''}QC}{NM}+\frac{\left( P^{'}Q \right)^{2}}{M^{2}N}>0$. | (S.10) |
| --- | --- | --- |

Combining (A.6) and $P^{'}<0$, we can also conclude that $A_{1}<0$, $A_{3}<0$ and

|  | $A_{1}B_{3}-B_{1}A_{3}=-\frac{P^{'}CM}{N}-\frac{P^{''}QC}{N^{2}}-\frac{P^{'}C}{N}>0$. | (S.11) |
| --- | --- | --- |

This also shows that

|  | $A_{3}B_{2}-B_{3}A_{2}>0$. | (S.12) |
| --- | --- | --- |

Substituting (A.7) - (A.9) into (A.3) and (A.4), we obtain $\frac{dQ}{dN}>0$ and $\frac{dM}{dN}>0$.

And, given that $\frac{dP}{dQ}<0$, we finally conclude that $\frac{dP}{dN}=\frac{dP}{dQ}\times\frac{dQ}{dN}<0$. □

- Cost reduction by specialization:

In this case, to ensure that the objective function attains its maxima at the critical point, the second order condition needs to be less than zero. That is, $SOC=\frac{{2P}^{'}}{N}+\frac{P^{''}Q}{N^{2}}-2\varphi^{'}-\varphi^{''}Q<0$. Intuitively, this means that the slope of the marginal revenue is steeper than the slope of the marginal cost.

To obtain well-behaved outcomes, the model also assumes that the marginal cost is downward sloping, meaning that $2\varphi^{'}+\varphi^{''}Q<0$. This condition further ensures that $\frac{{2P}^{'}}{N}+\frac{P^{''}Q}{N^{2}}<0$ given that $\frac{{2P}^{'}}{N}+\frac{P^{''}Q}{N^{2}}<2\varphi^{'}+\varphi^{''}Q$. Therefore, we have

$\frac{dQ}{dN}=\frac{\frac{Q}{N}(\frac{{2P}^{'}}{N}+\frac{P^{''}Q}{N^{2}})}{SOC}>0$.

Since $\frac{dM}{dQ}>0$, we have $\frac{dM}{dN}=\frac{dM}{dQ}\times\frac{dQ}{dN}>0$.

Finally, $\frac{dP}{dN}=P^{'}\frac{Q^{'}N-Q}{N^{2}}=P^{'}\times\frac{Q}{N^{2}}\times\frac{2\varphi^{'}+\varphi^{''}Q}{SOC}<0$. □

Note that, when certain conditions are satisfied, the comparative statics, $\frac{dM}{dN}$ and $\frac{dP}{dN}$, are the same for both the competition case and the specialization case.

*D. Description of the Most Commonly Outsourced Laboratory Tests*

Basic Metabolic Panel (CPT 80048): Blood panel. Tests for levels of calcium, carbon dioxide, chloride, creatinine, glucose, potassium, sodium, and urea nitrogen.

General Health Panel (CPT 80050): Blood panel. Tests for levels of albumin, bilirubin, calcium, carbon dioxide, chloride, creatinine, glucose, alkaline phosphate, potassium, protein, sodium, aspartate amino transferase, urea nitrogen, and thyroid stimulating hormone.

Electrolyte Panel (CPT 80051): Blood panel. Tests for levels of carbon dioxide, chloride, potassium, and sodium.

Lipid Panel (CPT 80061): Blood panel. Tests for levels of cholesterol, high-density lipoprotein (HDL), and triglycerides.

Hepatic Function Panel (CPT 80076): Blood Panel. Tests for levels of albumin, bilirubin, phosphate, protein, alanine amino transferase, and aspirate amino transferase.

Urinalysis with Microscopy (CPT 81001): Urine Panel. Done by dipstick, tests for levels of bilirubin, glucose, hemoglobin, ketones, leukocytes, nitrate, urobilinogen, as well as for the pH and specific gravity of the urine. Includes microscope examination.

Urinalysis without Microscopy (CPT 81003): Urine Panel. Done by dipstick, tests for levels of bilirubin, glucose, hemoglobin, ketones, leukocytes, nitrate, urobilinogen, as well as for the pH and specific gravity of the urine. Includes microscope examination.

Urine Test for Renal Disease or Diabetes (CPT 82043): Urine Panel. Tests for the level of albumin.

Test for Alpha-Fetoprotein Levels (CPT 82105): Blood Panel. Tests for the level of Alpha-Fetoprotein, a compound indicative of some forms of cancer.

Another Hepatic Function Panel (CPT 82150): Urine Panel. Tests for level of amylase.

1. $\mathrm{Prob}\left( \mathrm{Outsource}=1 \right)=\mathrm{Prob}\left[ \delta>P \right]=\mathrm{Prob}\left[ u>P-\delta\left( A \right) \right]=1-F\left[ P-\delta\left( A \right) \right]$. [↑](#footnote-ref-1)
2. This assumption is altered in the second scenario where we consider whether that the cost reduction of medical inputs is due to specialization. [↑](#footnote-ref-2)
3. To ensure that the function attains its maxima at the critical point, the second order condition, $\frac{{2P}^{'}}{N}+\frac{P^{''}Q_{j}}{N^{2}}$, should be less than zero (or $\frac{{2P}^{'}}{N}+\frac{P^{''}Q}{N^{2}M}<0$). This implies that the slope of the marginal revenue facing input suppliers is negative. To obtain well-behaved outcomes, the model also assumes that the marginal revenue is steeper than the demand function (Long and Soubeyran, 2000). That is, $\frac{{2P}^{'}}{N}+\frac{P^{''}Q_{j}}{N^{2}}<\frac{P^{'}}{N}$ , which can be simplified as $\frac{P^{'}}{N}+\frac{P^{''}Q_{j}}{N^{2}}<0$ (or $\frac{P^{'}}{N}+\frac{P^{''}Q}{N^{2}M}<0$). This is sensible for the medical input industry since provision of medical laboratory services usually involves a large investment in expensive medical equipment and the market hence does not entail free entry which would necessitate perfect competition. [↑](#footnote-ref-3)
4. Note that $M$ in Section 2.1 represents the number of medical labs, assuming each medical lab comprises only one employee given constant returns to scale. $M$ in Section 2.2 stands for the number of medical lab employees in this only one lab in the local area. Empirically, this variable can be captured in both cases by the amount of lab employment in the local area. [↑](#footnote-ref-4)
